# De Novo Lipogenesis in Adipose Tissue Is Associated with Course of Morbid Obesity after Bariatric Surgery

**DOI:** 10.1371/journal.pone.0031280

**Published:** 2012-02-23

**Authors:** Lourdes Garrido-Sánchez, Joan Vendrell, Diego Fernández-García, Victoria Ceperuelo-Mallafré, Matilde R. Chacón, Luis Ocaña-Wilhelmi, Juan Alcaide, Francisco J. Tinahones, Eduardo García-Fuentes

**Affiliations:** 1 CIBERDEM, Hospital Universitari Joan XXIII, IISPV, Tarragona, Spain; 2 Servicio de Endocrinología y Nutrición, Hospital Clínico Virgen de la Victoria, Málaga, Spain; 3 Ciber Fisiopatología Obesidad y Nutrición (CIBEROBN), Málaga, Spain; 4 Servicio de Cirugía, Hospital Clínico Virgen de la Victoria, Málaga, Spain; 5 Fundación IMABIS, Málaga, Spain; I2MC INSERM UMR U1048, France

## Abstract

**Objective:**

*De novo* lipogenesis is involved in fatty acid biosynthesis and could be involved in the regulation of the triglyceride storage capacity of adipose tissue. However, the association between lipogenic and lipolytic genes and the evolution of morbidly obese subjects after bariatric surgery remains unknown. In this prospective study we analyze the association between the improvement in the morbidly obese patients as a result of bariatric surgery and the basal expression of lipogenic and lipolytic genes.

**Methods:**

We study 23 non diabetic morbidly obese patients who were studied before and 7 months after bariatric surgery. Also, we analyze the relative basal mRNA expression levels of lipogenic and lipolytic genes in epiploic visceral adipose tissue (VAT) and abdominal subcutaneous adipose tissue (SAT).

**Results:**

When the basal acetyl-CoA carboxylase 1 (ACC1), acetyl-CoA synthetase 2 (ACSS2) and ATP citrate lyase (ACL) expression in SAT was below percentile-50, there was a greater decrease in weight (*P* = 0.006, *P* = 0.034, *P* = 0.026), body mass index (*P* = 0.008, *P* = 0.033, *P* = 0.034) and hip circumference (*P* = 0.033, *P* = 0.021, *P* = 0.083) after bariatric surgery. In VAT, when the basal ACSS2 expression was below percentile-50, there was a greater decrease in hip circumference (*P* = 0.006). After adjusting for confounding variables in logistic regression models, only the morbidly obese patients with SAT or VAT ACSS2 expression≥P50 before bariatric surgery had a lower percentage hip circumference loss (<P50) after bariatric surgery (SAT: *P* = 0.039; VAT: *P* = 0.033).

**Conclusions:**

A lower basal ACSS2, ACC1 and ACL expression, genes involved in *de novo* lipogenesis, is associated with a better evolution of anthropometric variables after bariatric surgery. Thus, the previous state of the pathways involved in fatty acid metabolism may have repercussions on the improvement of these patients.

## Introduction

Alterations in lipogenesis and lipolysis, mechanisms involved in the regulation of triglyceride storage, seem to be associated with obesity [1]. The lipogenesis pathway can be influenced by the supply of fatty acids-coenzyme A or glycerol phosphate, or by the activity of enzymes at later stages of triglyceride synthesis. Glycerol phosphorylation by glycerol kinase (GyK) and gluconeogenesis (GNG) are involved in the availability of glycerol phosphate. A regulatory step of GNG is the conversion of oxaloacetate to phosphoenolpyruvate, catalyzed by phosphoenolpyruvate carboxykinase (PEPCK). However, the role of GyK and PEPCK seems to be limited in adipose tissue and is not well known in human obesity [2]. Dysregulation of PEPCK might influence lipid deposition and therefore contribute to obesity and diabetes [3], [4].

In vivo studies have suggested that one possible site for regulation of fatty acid synthesis from acetate is at the level of the enzyme acetyl-CoA synthetase (ACSS) [5]. Mammals have two types of ACSS: mitochondrial ACSS1 and cytosolic ACSS2 [6]. ACSS1 is a mitochondrial enzyme that produces acetyl-CoA for oxidation in the tricarboxylic acid cycle [6]. Mitochondrial acetyl-CoA produced by ACSS1 becomes citrate, which is then preferentially exported to the cytosol and cleaved into acetyl-CoA and oxaloacetate by ATP citrate lyase (ACL) [7]. This acetyl-CoA can be used for lipid synthesis [8]. ACL is a critical enzyme that regulates the initial step in lipid biosynthesis and is considered a key enzyme linking glucose metabolism to lipid synthesis. ACSS2 is a cytosolic enzyme that catalyzes the formation of acetyl-coenzyme A from coenzyme A and acetate [5], [9], an essential molecule utilized in various metabolic pathways including fatty acid [5], [6], [8], [9]. Steiner et [10] suggested the role of this enzyme in the control of fatty acid synthesis based on studies in brown adipose tissue homogenates. In addition, acetyl-CoA carboxylase 1 (ACC1) mediates the conversion from acetyl-CoA to malonyl-CoA. This molecule produced by ACC1 is thought to be used selectively as a substrate for *de novo* lipogenesis [11]. Although *de novo* lipogenesis can account for only a small fraction of total triglyceride flux in adipose tissues, under certain conditions, such as low fat ingest, may have some relevance. Its importance in the determination of adipose tissue mass has not been resolved [12]. Changes in lipolysis might also shift the balance of fatty acid flux and modulate fat mass. A modest energetic imbalance over time can predispose to obesity. Conversely, fat loss by dieting is a slow process and the involvement of the lipogenic and lipolytic enzymes is not fully understood.

Bariatric surgery is almost the only effective strategy for treating morbidly obese patients. However, morbidly obese patients do not all lose the same weight after the same type of bariatric surgery, nor do their accompanying disorders improve the same or at the same rates. This variation between patients in metabolic progress may depend on the metabolic state of their adipose tissue before surgery, thus conditioning their metabolic response to the operation. After bariatric surgery weight loss is mostly produced by a reduction in the amount of stored fat. When lipid absorption is limited (i.e. starvation or biliopancreatic diversion (BDP)), fatty acids and glycerol come from lipolysis of adipose tissue and other tissue reserves. The previous state of the different pathways involved in fatty acid metabolism may have repercussions on the improvement of these patients.

Recently, our group has shown an upregulation of genes facilitating triglyceride/fatty acid cycling and a reduction in the genes involved in *de novo* synthesis of fatty acids in morbid obesity [1]. However, the association between lipogenic and lipolytic genes and the evolution of morbidly obese subjects after bariatric surgery remains unknown. In the present prospective study we analyzed the association between the anthropometric and biochemical changes produced in morbidly obese patients in the opposite situation, i.e., an improvement in these variables as a result of bariatric surgery, with the basal expression of lipogenic and lipolytic genes.

## Results

### Bariatric surgery improved the anthropometric and biochemical characteristics

Table 1 summarizes the characteristics of the morbidly obese patients before and 7 months after their bariatric surgery. Seven months after surgery, the anthropometric and biochemical variables studied improved significantly. Waist and hip circumferences, weight, BMI and HOMA-IR all decreased (18.2±8.8%, 17.6±6.7%, 26.7±10.5%, 26.7±10.5% and 65.3±20.6%, respectively) (p<0.001).

**Table 1:** Anthropometric and biochemical variables in the morbidly obese patients before and after surgery.

	Morbidly obese before surgery	Morbidly obese after surgery
**N (men/women)**	23(9/14)	-
**Age (years)**	39.8±10.3	-
**Weight (kg)**	158.0±28.3	114.4±18.68^c^
**BMI (kg/m^2^)**	57.4±7.1	41.70±6.59^c^
**Waist (cm)**	146.1±23.5	118.5±15.09^c^
**Hip (cm)**	160.3±16.7	132.2±12.69^c^
**Glucose (mM)**	5.67±1.01	4.70±0.51^b^
**Cholesterol (mM)**	4.99±0.76	3.39±0.60^c^
**HDL cholesterol (mM)**	1.15±0.38	0.98±0.23^a^
**LDL cholesterol (mM)**	3.06±0.85	1.87±0.047^c^
**VLDL cholesterol (mM)**	0.65±0.43	0.54±0.20
**Triglycerides (mM)**	1.45±0.88	1.21±0.43
**Free fatty acids (mM)**	0.614±0.287	0.475±0.168^a^
**Insulin (pM)**	31.5±18.7	10.74±4.51^c^
**HOMA-IR**	8.13±5.90	2.21±1.02^c^

BMI: body mass index; LDL: low density lipoprotein; VLDL: very low density lipoprotein; HOMA-IR: homeostasis model assessment of insulin resistance index. The results are given as the mean ± SD.

a P<0.05;

b P<0.01;

c P<0.001.

### A lower basal mRNA expression of *de novo* lipogenesis genes is associated with a better evolution of anthropometric variables after bariatric surgery

**Figure 1:**
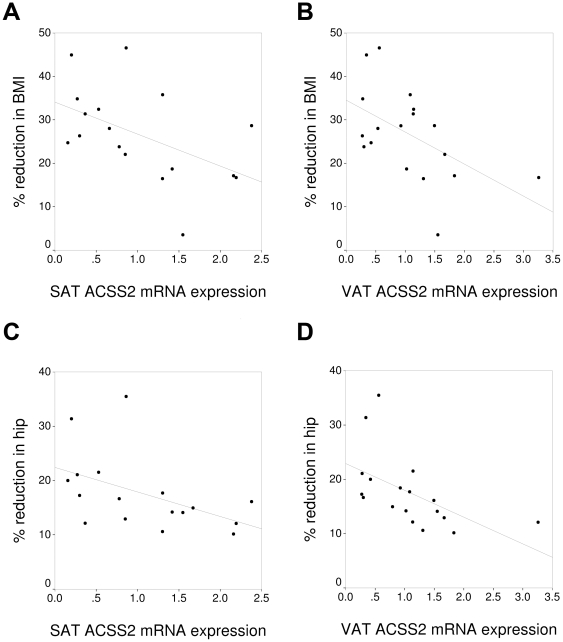
Association between the expression of acetyl-CoA synthetase 2 (ACSS2) and the percentage of reduction in the body mass index (BMI) in subcutaneous (A) and visceral adipose tissue (B), and the hip circumference in SAT (C) and VAT (D). BMI: body mass index.

**Figure 2:**
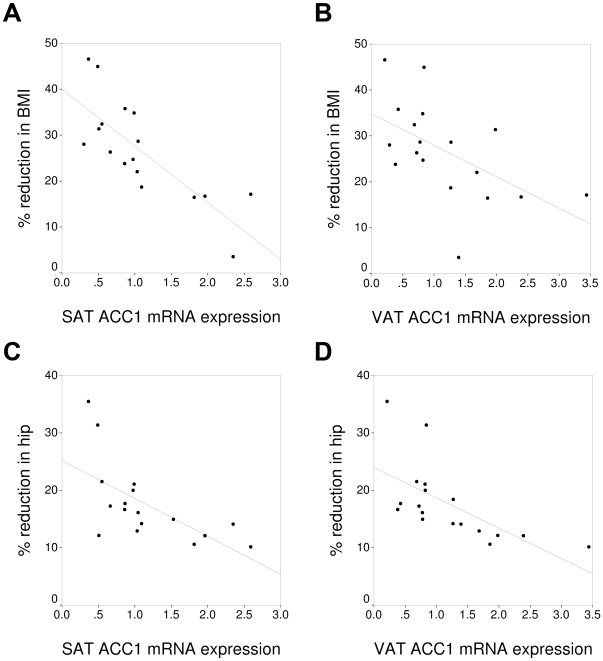
Association between the expression of acetyl-CoA carboxylase 1 (ACC1) and the percentage of reduction in the body mass index (BMI) in subcutaneous (A) and visceral adipose tissue (B), and the hip circumference in SAT (C) and VAT (D). BMI: body mass index.

The basal expression of ACSS2 (Figure 1) and ACC1 (Figure 2) in VAT and SAT correlated negatively with the percentage decrease in weight, BMI and hip circumference after bariatric surgery (Table 2). The basal expression of PEPCK1 in SAT correlated negatively with the percentage reduction in weight, BMI and hip circumference (Table 2). The percentage reduction in FFA correlated significantly with the basal expression of ACC1 and ACSS2 in SAT (Table 2). The percentage reduction in HOMA-IR only correlated significantly with the basal expression of PEPCK1 and ACL in SAT (Table 2). The expression of the other genes studied in SAT and VAT (PPARγ, DGAT1, AQP7, GyK, ATGL, HSL and perilipin) did not correlate significantly with the change in the biochemical variables studied after bariatric surgery (data not shown).

**Table 2:** Significant correlations (*p*) found between basal mRNA expression and the anthropometric and biochemical variables studied.

		% reduction in weight	% reduction in hip circumference	% reduction in BMI	% reduction in FFA	% reduction in HOMA-IR
**ACC1**	**SAT**	−0.732 (0.001)	−0.624 (0.033)	−0.731 (0.001)	−0.459 (0.055)	Ns
	**VAT**	−0.522 (0.023)	−0.639 (0.010)	−0.576 (0.014)	Ns	Ns
**ACSS2**	**SAT**	−0.521 (0.039)	−0.566 (0.018)	−0.526 (0.052)	−0.519 (0.027)	Ns
	**VAT**	−0.623 (0.004)	−0.619 (0.015)	−0.710 (0.001)	Ns	Ns
**ACL**	**SAT**	Ns	Ns	Ns	Ns	−0.592 (0.006)
	**VAT**	Ns	Ns	Ns	Ns	Ns
**PEPCK1**	**SAT**	−0.529 (0.034)	−0.749 (0.002)	−0.529 (0.049)	Ns	−0.492 (0.038)
	**VAT**	Ns	Ns	Ns	Ns	Ns

BMI: body mass index; FFA: free fatty acids; HOMA-IR: homeostasis model assessment of insulin resistance index; ACC1: acetyl-coenzyme carboxylase 1; ACSS2: acetyl-CoA synthetase short-chain family member 2; ACL: ATP citrate lyase; PEPCK1: phosphoenolpyruvate carboxykinase 1; SAT: subcutaneous adipose tissue; VAT: visceral adipose tissue; Ns: not significant. Correlation analyses were made with general linear models to adjust by age, gender, and baseline body weight and BMI as independent covariate.

Once we had seen the association between the basal expression of ACC1, ACSS2, ACL and PEPCK1 and the improvement of the different anthropometric variables after bariatric surgery, the patients were classified according to the 50^th^ percentile (<P50 or ≥P50) of the distribution of the basal expression of each of these three genes, both in SAT (Table 3) and in VAT. The association between the expression of these genes and the improvement of the different anthropometric variables was mainly seen in SAT. Table 3 shows the percentage reduction in weight, BMI, hip circumference, FFA and HOMA-IR according to the expression of these genes in SAT. We see that when the expression of ACC1, ACSS2 and ACL was below P50, there was a greater percentage reduction in weight (p = 0.006, p = 0.034, p = 0.026, respectively), BMI (p = 0.008, p = 0.0033, p = 0.034, respectively), hip circumference (p = 0.033, p = 0.021, p = 0.083, respectively), levels of FFA (p = 0.536, p = 0.019, p = 0.161, respectively) and HOMA-IR (p = 0.478, p = 0.606, p = 0.002, respectively) after bariatric surgery (Table 3). When the expression of PEPCK1 was below P50, there was a greater percentage reduction in weight (p = 0.049) after bariatric surgery (Table 3). No significant differences were found for the other genes studied in SAT (PPARγ, DGAT1, AQP7, GyK, ATGL, HSL and perilipin) (data not shown).

**Table 3:** Reduction in the anthropometric and biochemical variables according to the 50^th^-percentile of the expression of different genes in subcutaneous adipose tissue.

		% reduction in weight	% reduction in hip circumference	% reduction in BMI	% reduction in FFA	% reduction in HOMA-IR
**ACC1**	**<P50**	33.6±8.3^b^	21.7±8.5^a^	33.6±8.3^b^	24.9±38.7	71.8±17.7
	**≥P50**	20.7±8.4	14.6±3.6	20.3±8.8	−23.2±118.1	59.5±22.9
**ACSS2**	**<P50**	31.5±8.5^a^	20.9±7.8^a^	31.5±8.5^a^	37.7±31.2^a^	66.5±20.3
	**≥P50**	20.2±9.6	13.7±2.6	19.5±10.2	−46.7±121.7	63.0±23.5
**ACL**	**<P50**	27.4±10.8^a^	19.6±10.3	27.2±11.3^a^	7.1±58.8	73.7±12.7^b^
	**≥P50**	20.7±4.0	18.0±5.3	20.7±4.0	−42.5±39.6	50.2±16.6
**PEPCK1**	**<P50**	30.6±6.5^a^	19.1±5.6	30.6±6.5	24.6±36.5	72.8±18.1
	**≥P50**	22.3±12.3	16.1±7.9	22.0±13.1	−27.7±123.3	58.7±22.2

BMI: body mass index; FFA: free fatty acids; HOMA-IR: homeostasis model assessment of insulin resistance index; ACC1: acetyl-coenzyme carboxylase 1; ACSS2: acetyl-CoA synthetase short-chain family member 2; ACL: ATP citrate lyase; PEPCK1: phosphoenolpyruvate carboxykinase 1;

a P<0.05;

b P<0.01;

c P<0.001: significant differences between the percentage reduction in the different variables according to the 50^th^ percentile of the expression of each of the genes.

In the VAT when the basal levels of ACSS2 expression were below P50 the percentage reduction in hip circumference was greater (21.9±7.4 vs. 14.1±3.4; p = 0.006). No significant differences were found in the percentage reduction in weight, BMI, HOMA-IR or FFA (data not shown). For the other genes studied in VAT (PPARγ, ACC1, ACL, PEPCK1, DGAT1, AQP7, GyK, ATGL, HSL and perilipin) no significant differences were found in the percentage reduction in the hip circumference, weight, BMI, HOMA-IR or FFA (data not shown).

The contribution of the ACC1, ACSS2, ACL and PEPCK1 gene expression to the improvement experienced after bariatric surgery was calculated from a contingency table in which the exposure variable was the level of gene expression above or below the 50th percentile of the morbidly obese subjects before bariatric surgery (<P50 or ≥P50) and the dependent variable was the percentage reduction in the study variable (weight, BMI, waist and hip circumferences, HOMA-IR or FFA) after the seven months of follow-up (<P50 or ≥P50).

Morbidly obese patients with SAT ACC1 expression levels below the 50th percentile (<P50) before bariatric surgery had a relative risk (RR) of 4.375 (95% CI: 1.232–15.321) of having a greater percentage weight loss (≥P50) and a RR of 4.286 (95% CI: 1.196–15.325) of having a greater percentage hip circumference loss (≥P50) after bariatric surgery than those with SAT ACC1 expression levels ≥P50. In VAT, no significant results were found with ACC1 expression levels (data not shown).

Morbidly obese patients with SAT ACSS2 expression levels ≥P50 before bariatric surgery had a higher risk (RR: 3.938, 95% CI: 1.232–15.321) of having a lower percentage hip circumference loss (<P50) after bariatric surgery than those with ACSS2 expression levels <P50. Morbidly obese patients with VAT ACSS2 expression levels <P50 before bariatric surgery had a higher risk (RR: 4.375, 95% CI: 1.232–15.531) of having a greater percentage hip circumference loss (≥P50) after bariatric surgery than those with VAT ACSS2 expression levels ≥P50.

Morbidly obese patients with SAT ACL expression levels ≥P50 before bariatric surgery had a higher risk (RR: 2.852, 95% CI: 1.022–7.958) of having a lower percentage HOMA-IR loss (<P50), a higher risk (RR: 2.406, 95% CI: 1.055–5.488) of having a lower percentage weigh loss (<P50) and a higher risk (RR: 2.917, 95% CI: 1.092–7.789) of having a lower percentage BMI loss (<P50) after bariatric surgery than those with ACL expression levels <P50. In VAT, no significant results were found with ACL expression levels (data not shown). With PEPCK1 expression levels, no significant results were found in SAT or VAT (data not shown).

However, after adjusting for age, gender, weight and BMI in logistic regression models, only the morbidly obese patients with SAT or VAT ACSS2 expression levels≥P50 before bariatric surgery had a lower percentage hip circumference loss (<P50) after bariatric surgery (SAT: *P* = 0.039; VAT: *P* = 0.033).

Given the relation between the expression of ACC1, ACCS2 and ACL and the anthropometric evolution of the morbidly obese patients after their surgery, we analyzed the basal anthropometric and biochemical variables according to the basal expression of these enzymes (<P50 or ≥P50) in SAT and VAT. We found no significant differences in any of the variables between the two groups (ACC1<P50 versus ACC1≥P50; ACSS2<P50 versus ACSS2≥P50; ACL<P50 versus ACL≥P50) (data not shown), with the exception of FFA. Those morbidly obese patients with a basal expression of SAT ACSS2<P50 had a higher basal FFA concentration than those with SAT ACSS2≥P50 (0.760±0.233 mM vs. 0.485±0.290 mM, p = 0.024).

## Discussion

The mayor finding of this study is that *de novo* lipogenesis of adipose tissue seem to be involved in the medium-term evolution of anthropometric variables after bariatric surgery. To date, no studies exist that show the relationship between adipose tissue gene expression and weight loss after bariatric surgery. In this study we see the association between baseline lipogenic and lipolytic genes expression levels with change of anthropometric variables after bariatric surgery. Previously, we have study the association between basal expression level and basal anthropometric variables [1]. Our results, like those of others [13], [14], show that anthropometric and biochemical variables improve significantly in morbidly obese patients after bariatric surgery. After BPD, weight loss is primarily due to a selective malabsorption [15]. Nevertheless, anthropometric variables improve more in some patients than others. Though various physiological factors and other mechanisms are involved in this weight loss, they are not all fully understood. The lipogenic capacity of adipose tissue could be a variable to consider, as seen from this study. The lower basal expression of the genes involved in *de novo* fatty acid synthesis was associated with a better short-term anthropometric evolution in these patients after bariatric surgery.

After bariatric surgery, predominantly lipolytic state, fatty acid re-esterification acts as a retro-control pathway to reduce FFA release. In the lipolytic state, GNG is the main provider of G3P, and the key enzyme of GNG is PEPCK1 [2], [16]. In previous studies, adipose tissue GNG has been associated with obesity [1], [3], [4]. The negative association found between PEPCK1 and variables related to weight loss indicates that PEPCK1 could, to a certain extent, be associated with the control of adipose tissue triglyceride storage in morbidly obese patients. A lower GNG, pathway that generates G3P to lipogenesis, would be directly related with a greater weight loss.

Biliopancreatic diversion produces lipid malabsorption [15], with reduced levels of plasma FFA and interruption of the Randle cycle. In the biosynthesis of fatty acids, the regulation of the first steps in *de novo* lipogenesis may be of great importance. ACC1 is predominantly expressed in lipogenic tissues, such as adipose tissue [17]. ACC1 mediates the initial step of the fatty acid synthesis, suggesting that ACC1 could play a crucial role in the regulation of lipogenesis. As other studies [17], [18], our data support a potential role for ACC1 in the development of obesity. The expression of ACC1 is associated with variables related with weight loss. Inactivation of ACC1 would result in inhibition of fatty acid and malonyl-CoA synthesis, reduced lipid accumulation in adipose tissue and stimulation of fatty acid oxidation [19].

Previous studies suggest that ACCS2 has an important role in the control of fatty acid synthesis in adipose tissue [10]. The present study shows that morbidly obese patients with a lower expression of ACSS2 in SAT have higher plasma levels of FFA. It is also in agreement with studies showing that a high fat diet [10], fasting or addition of fatty acids in cultured cells [9] produce inhibition of ACSS2 activity. In all these cases the fatty acids increase. Our results also show that those morbidly obese persons who have a lower basal expression of ACSS2 in SAT experience a greater percentage reduction in plasma FFA after bariatric surgery. This greater reduction may be the consequence of lower synthesis of lipids from acetate, given the lower expression of ACSS2. As we showed, this lower expression of ACSS2 would result in a greater reduction in adipose tissue after bariatric surgery. The current study clearly demonstrates that a lower basal expression of the ACSS2 gene in VAT and SAT is associated with a greater loss of weight, BMI and hip circumference after BPD.

ACL catalyzes the production of cytosolic acetyl-CoA and oxaloacetate from citrate, thereby linking cellular glucose catabolism and *de novo* lipid synthesis. As we showed, a lower expression of ACL in SAT would result in a greater reduction of insulin resistance, weight and BMI after bariatric surgery. As other studies [20], our data support a potential role for ACL in the development of obesity. However, contradictory studies exist on the role of ACL in obesity [20]–[22]. Our results demonstrate that ACL is associated with variables related with weight loss. This enzyme could play a crucial role in the regulation of lipogenesis and could be involved in the improvement of insulin resistance at the whole-body level, as other authors suggest [23]. Although of greater complexity in the regulation of cell lipid biosynthesis, ACSS2, ACC1 and ACL are sites of control of the regulation in the overall process of fatty acid synthesis. While the importance of the role of fatty acids in the regulation of obesity is well known, our findings go beyond this. We show the possible role of the regulation of the first steps in *de novo* synthesis on the course of anthropometric variables after bariatric surgery.

Previous results suggest differing degrees of involvement of VAT and SAT in obesity and insulin resistance [1]. Our results also suggest that the degree of involvement of VAT and SAT in the evolution of anthropometric variables after bariatric surgery differs. Since BPD causes malabsorption in small intestine, it is possible that effects of this malabsorption could be enhanced in VAT. However, basal SAT expression levels of genes involved in *de novo* lipid synthesis show a higher involvement than VAT expression levels in the changes produced in the anthropometric variables as a result of bariatric surgery. For example, VAT ACL and PEPCK gene expression is not associated with the changes produced in the anthropometric variables. Recently has been suggested that increasing weight loss attenuates the preferential loss of VAT vs. SAT fat [24]. Also, changes in the foregut incretin pattern, the reduction in free fatty acids, or other changes in the gastrointestinal tract after BDP could differently affect to VAT and SAT. These two adipose tissues are metabolically different and the response to hormones differ [25], [26]. These differences may partly be explained by the regional variations in the response to the action of insulin [25], [27].

The results of this study suggest that inactivation of *de novo* lipogenesis in adipose tissue, which would reflect good adaptation of the adipose tissue to the excess of fat and FFA, may influence the loss of adipose tissue after treatment. When the absorption of fat decreases, as a consequence of bariatric surgery, adipose tissue is obliged to increase lipolysis. Those morbidly obese patients whose basal *de novo* lipogenesis is more inactivated are better suited metabolically to experience a more drastic reduction in fat depots. However, we were unable to measure the amount of fat lost nor the sites where it was lost by more exact methods, such as computerized tomography or magnetic resonance imaging. More complete studies are now required to confirm the physiological role of these enzymes in the regulation of cellular energy metabolism. The benefits and risks of modulating a single component of this complex system remain to be explored, with vigilance for possible unpredicted effects at sites remote from adipose tissues.

In conclusion, *de novo* lipogenesis seem to be involved in the medium-term evolution of several anthropometric variables after bariatric surgery. The basal status of the enzymes involved in *de novo* lipogenesis may condition the response of morbidly obese persons to this surgery. Different markers are required to help explain the enormous variability in the evolution of obese persons after bariatric surgery or other therapeutic interventions. Also, These markers will enable more efficient selection of the type of treatment in such a prevalent disease as obesity, as well as identification of those patients who will respond better.

## Materials and Methods

### Subjects

The study included 23 non diabetic morbidly obese patients (BMI 57.4±5.2 Kg/m^2^) who were studied before and 7 months after bariatric surgery [28]–[30]. All the patients underwent open biliopancreatic diversion of Scopinaro (BPD). Patients were excluded if they had type 2 diabetes mellitus, cardiovascular disease, arthritis, acute inflammatory disease, infectious disease, or were receiving drugs that could alter the lipid profile or the metabolic parameters at the time of inclusion in the study. The weight of all the persons had been stable for at least one month and none had renal involvement. All participants gave written informed consent and the study was reviewed and approved by the Ethics and Research Committee of Virgen de la Victoria Clinical University Hospital, Malaga, Spain.

### Laboratory measurements

Blood samples were collected after a 12-hour fast, before and 7 months after bariatric surgery [28]–[30]. The serum was separated and immediately frozen at −80°C. Serum biochemical parameters were measured in duplicate. Serum glucose, cholesterol, HDL cholesterol, triglycerides (Randox Laboratories Ltd., Antrium, UK) and free fatty acids (FFA) (WAKO Chemicals, Richmond, VA) were measured by standard enzymatic methods. LDL and VLDL cholesterol were calculated by Friedewald's formula. The insulin was analyzed by an immunoradiometric assay (BioSource International, Camarillo, CA), showing a 0.3% cross-reaction with proinsulin. The homeostasis model assessment of insulin resistance index (HOMA-IR) was calculated from fasting insulin and glucose with the following equation: HOMA-IR = fasting insulin (µIU/mL)×fasting glucose (mol/L)/22.5. The percentage change in the different anthropometric and biochemical variables as a consequence of the bariatric surgery was calculated as: (basal variable−variable at 7 months)×100/basal variable [31].

### Visceral and subcutaneous adipose tissue mRNA

We analyzed the relative basal mRNA expression levels of lipogenic and lipolytic genes in epiploic visceral adipose tissue (VAT) and abdominal subcutaneous adipose tissue (SAT). Five grams of VAT and SAT were obtained during bariatric surgery in the morbidly obese patients [1], [32]. The biopsy samples were washed in physiological saline and immediately frozen in liquid nitrogen. Biopsy samples were maintained at −80°C until analysis. Frozen adipose tissue was homogenized with an Ultra-Turrax 8 (Ika, Staufen, Germany). Total RNA was extracted using RNeasy lipid tissue midi kit (QIAGEN Science, Hilden, Germany), and total RNA was treated with 55URNasefree deoxyribonuclease (QIAGEN) following the manufacturer's instructions. The purity of the RNA was determined by the absorbance260/absorbance280 ratio on the Nanodrop. The integrity of total purified RNA was checked by denaturing agarose gel electrophoresis and ethidium bromide staining. Total RNA was reverse transcribed to cDNA by using a high-capacity cDNA reverse transcription kit with RNase inhibitor (Applied Biosystems, Foster City, CA). The cDNA was used for quantitative real-time PCR with duplicates. We analyzed the relative basal mRNA expression levels of peroxisome proliferator-activated receptor-ã (PPARã) (Hs00234592_m1, RefSeq. NM_005037.5, NM_015869.4, NM_138711.3 and NM_138712.3), acyl Coenzyme A:cholesterol acyltransferase (DGAT1) (Hs00201385_m1, RefSeq. NM_012079.4), aquaporin 7 (AQP7) (Hs00357359_m1, RefSeq. NM_001170.1), acetyl-CoA carboxylase 1 (ACC1) (Hs00167385_m1, RefSeq. NM_198834.1, NM_198836.1, NM_198837.1, NM_198838.1 and NM_198839.1), acetyl-CoA synthetase short-chain family member 2 (ACSS2) (Hs00218766_m1, RefSeq. NM_001076552.2, NM_018677.3 and NM_028046.1), ATP citrate lyase (ACL) (Hs00982738_m1, RefSeq. NM_001096.2 and NM_198830.1), phosphoenolpyruvate carboxykinase (PEPCK) (Hs00159918_m1, RefSeq. NM_002591.3), glycerol kinase (GyK) (Hs02340011_g1, RefSeq. NM_000167.4 and NM_203391.2), adipose triglyceride lipase (ATGL) (Hs00386101_m1, RefSeq. NM_020376.3), hormone-sensitive lipase (HSL) (Hs00193510_m1, RefSeq. NM_005357.2), and perilipin (Hs00160173_m1, RefSeq. NM_001145311.1 and NM_002666.4). We choose these genes because, previously, we have study the association between these lipogenic and lipolitic genes with basal anthropometric and biochemical variables [1]. The cycle threshold (Ct) value for each sample was normalized with the expression of PPIA (Hs99999904_m1). There is no variation in the expression of this housekeeping gene in the condition tested. Real-time quantitative PCR was performed on a 7900HT Fast real-time PCR system using commercial Taqman assays (Applied Biosystems) [33]. SDS software 2.3 and RQ Manager 1.2 (Applied Biosystems) were used to analyze the results with the comparative Ct method (2−ΔΔCt). All data were expressed as an n-fold difference relative to the calibrator (a mixture of the SAT and VAT tissues was used as calibrator sample).

### Statistical analysis

The statistical analysis was done with SPSS (Version 11.5 for Windows; SPSS, Chicago, IL). The Wilcoxon test for paired samples was used to analyze the differences in the biological variables between the first and the second sampling, 7 months after the operation. General lineal models were calculated to estimate the correlations between variables. The Mann-Whitney test was used to compare the results of the two groups of morbidly obese patients, defined according to whether the expression of the lipogenic enzymes analyzed (ACSS2 and ACC1) was below the 50^th^ percentile (<P50) or above it (≥P50). We choose 50th percentile to have two groups with similar number of patients in each group. The contribution of the gene expression to the improvement seen after bariatric surgery was calculated from a contingency table in which the exposure variable was the level of gene expression above or below the 50th percentile of the morbidly obese subjects before bariatric surgery and the dependent variable was percentage reduction in the study variable (weight, BMI, waist and hip circumferences, HOMA-IR or FFA) over the seven months of follow-up. Statistical significance was calculated from the χ2 test. Logistic regressions were used to determine the contribution of the gene expression to the improvement seen after bariatric surgery, after adjusting for age, gender, weight and BMI. Values were considered to be statistically significant when the *P*≤0.05. The results are given as the mean ± SD.
